# Genome editing in Sub-Saharan Africa: a game-changing strategy for climate change mitigation and sustainable agriculture

**DOI:** 10.1080/21645698.2024.2411767

**Published:** 2024-10-31

**Authors:** Peter Amoah, Abdoul-Razak Oumarou Mahamane, Moise Hubert Byiringiro, Neo Jeremiah Mahula, Nyimasata Manneh, Yetunde Ruth Oluwasegun, Abebawork Tilahun Assfaw, Hellen Mawia Mukiti, Abubakar Danlami Garba, Felicity Kido Chiemeke, Omena Bernard Ojuederie, Bunmi Olasanmi

**Affiliations:** a Plant Breeding Programme, Pan African University Life and Earth Sciences Institute (Including Health and Agriculture), Ibadan, Nigeria; b Department of Irrigated Crop, National Agricultural Research Institute of Niger (INRAN), Niamey, BP, Niger; c Department of Biological Sciences, Biotechnology Unit, Faculty of Science, Kings University, Ode-Omu, Nigeria; d Food Security and Safety Focus Area, Faculty of Natural and Agricultural Sciences, North-West University, Mmabatho, South Africa; e Department of Crop and Horticultural Science, Faculty of Agriculture, University of Ibadan, Ibadan, Nigeria

**Keywords:** Climate resilience, CRISPR/Cas9, crop Improvement, food security, gene editing, plant breeding, sustainable agriculture

## Abstract

Sub-Saharan Africa’s agricultural sector faces a multifaceted challenge due to climate change consisting of high temperatures, changing precipitation trends, alongside intensified pest and disease outbreaks. Conventional plant breeding methods have historically contributed to yield gains in Africa, and the intensifying demand for food security outpaces these improvements due to a confluence of factors, including rising urbanization, improved living standards, and population growth. To address escalating food demands amidst urbanization, rising living standards, and population growth, a paradigm shift toward more sustainable and innovative crop improvement strategies is imperative. Genome editing technologies offer a promising avenue for achieving sustained yield increases while bolstering resilience against escalating biotic and abiotic stresses associated with climate change. Clustered Regularly Interspaced Short Palindromic Repeats/CRISPR-associated protein (CRISPR/Cas) is unique due to its ubiquity, efficacy, alongside precision, making it a pivotal tool for Sub-Saharan African crop improvement. This review highlights the challenges and explores the prospect of gene editing to secure the region’s future foods.

## Introduction

The world’s citizenry is expected to rise, surpassing 9.7 billion by 2050 and possibly reaching a peak of over 10.4 billion by the mid-2080s, according to demographic forecasts.^1,2^ This necessitates a significant transformation of food production systems to address the challenge of sustaining this burgeoning population, ensuring sufficient food and industrial raw material production to meet a growing population amidst a complex web of constraints.^3^ These constraints encompass a multitude of political, economic, and environmental factors, including those exacerbated by climate change.^4^

Climate change stands as the 21st century’s most significant environmental threat, impacting the entire planet.^5^ Increasing temperatures alongside changed rainfall trends demonstrably impact environments, wildlife, and human societies globally.^6,7^ This unprecedented shift translates into substantial environmental damage and critical challenges for agricultural production.^8–10^ Food security for a growing global population faces significant dangers due to a confluence of factors: increased pest and disease emergence, escalating environmental stressors (famine, floods, and pollution), water resource depletion, and shrinking arable land.^11^ With continued temperature rise and precipitation irregularity, these repercussions are projected to worsen in times to come.^5,12^

Despite existing climate change research,^13^ a critical gap exists especially in developing countries, where financial limitations often hinder the adoption of advanced adaptation strategies.^3^ The impact of climate change is demonstrably heterogeneous across nations^11^ (Milesi).^15^ This heterogeneity is further amplified by the effectiveness of implemented adaptation and mitigation efforts, heavily influenced by available financial resources.^14,16,17^

Sub-Saharan Africa’s (SSA) heavy reliance on a climate-sensitive agricultural system renders its economies perpetually vulnerable.^18,19^ Climate change is predicted to exacerbate this disparity compared to developed nations, potentially widening the income gap.^20^ SSA is at significant risk from climate change because of its reliance on agriculture and forestry, mild temperature, unpredictable rainfall, and little ability to adapt. Rising temperatures are projected to shorten growing seasons for existing crops, further jeopardizing food security.^21^ Elevated temperatures pose a significant threat to crop yields, particularly during critical growth stages like flowering.^22^ The Intergovernmental Panel on Climate Change (IPCC^23^) anticipates in its 2021 report that SSA will be exceptionally vulnerable by 2100, with projected crop losses ranging from 5% to 15% of Gross Domestic Product (GDP). In a worst-case scenario, the average output of staple grain crops in SSA is anticipated to decline substantially.

Traditional crop improvement strategies in SSA have primarily relied on Quantitative Trait Loci (QTL) mapping and marker-assisted selection, leveraging phenotypic and pedigree data for breeding value selection. While historically successful, these methods are often resource-intensive, laborious, and yield variable results.^24^ Conversely, advancements in biotechnology have revolutionized plant breeding in developed nations. However, their application in SSA remains hampered by weak economic infrastructure, limited research funding and facilities, scarce technical expertise, unclear regulatory framework and biosafety protocols which causes delay, public skepticism, lack of political will and commitment to biotechnology, and inadequate agricultural policies. Consequently, fostering an environment conducive to deploying modern technologies for crop improvement in SSA appears exceptionally challenging.

The development of genomics and gene editing methodologies has generated a plethora of opportunities and prospective solutions for enhancing agricultural genetic traits.^25^ The advancement of modern instruments such as next-generation sequencing (NGS) techniques, cutting-edge genotyping arrays, genome mapping, and genomic selection technologies has significantly accelerated the crop breeding processes and created numerous new opportunities for genomics and genome editing approaches.^26,27^ These methods enable the creation of elite gene pools by effectively harnessing the untapped diversity found in crop wild relatives.^28^ Genes can now be precisely edited using modern gene editing tools to create new kinds with desired traits.^29,30^ Utilizing DNA markers and genome editing techniques in crop breeding is considered a highly effective approach to sustain consistent productivity growth in the face of escalating biotic and abiotic pressures, as well as heightened climate vulnerability.^31^

Advanced genomic tools like meganucleases, zinc-finger nucleases (ZFNs), transcription activator-like effector nucleases (TALENs), and clustered regularly interspaced short palindromic repeats/CRISPR-associated protein (CRISPR/Cas) offer precision for targeted genetic modifications in crop breeding.^32^ This facilitates the swift development of new cultivars with the preferred characteristics for climate change resilience of more than 9.7 billion population by 2050 (Ritchie et al., 2023), without the risk of introducing unexpected repercussions such as inbreeding depression and probable-off target impacts often associated alongside traditional methods like backcrossing and transgenesis. Three different forms of site-directed nuclease (SDN) genome editing are distinguished by regulatory mechanisms. SDN-1 makes minor adjustments to the target location using template-guided repair by homologous recombination, and SDN-2 replaces a particular DNA sequence in the genome. Similar to SDN-2, SDN-3 introduces bigger genetic components, such as entire genes.^33,34^ This review dissects the multifaceted challenges hindering SSA agriculture, exacerbated by climate change’s detrimental effects on key crops, the burgeoning utilization of gene editing for crop improvement and climate change mitigation, and examines the future potential of genome editing technology for food and nutrition security in the region.

## Effect of Climate Change on Agriculture in SSA

Most of African countries population relies on agriculture for livelihoods and income. The agricultural sector plays a substantial role, contributing between 20% and 30% to the overall GDP.^35^ The majority of SSA population relies on rain-fed agriculture,^36^ which constitutes approximately 97% of the total cultivated land, leading to considerable seasonal variability in agriculture.^37^

The effect of climate change on agriculture in SSA is of particular concern due to the region's vulnerability to multiple stresses and high temperature combined with limited adaptation capacities, therefore exacerbating the vulnerability of the population.^38–41^ As an illustration, elevated temperatures are anticipated to shorten the growth period for existing crop varieties in both arid and semi-arid zones.^21,42^ Crop yields are directly affected by both the quantity and variability of rainfall^42–44^; nevertheless, adapting to changing rainfall patterns becomes a challenge due to uncertainty regarding magnitude of rainfall projections and distribution.^42,45–47^ Given the fast rate of climate change and the escalating scale of probable consequences, agricultural adaptation is urgently needed, and comprehensive and resilient adaptation strategies are necessary, including the use of biotechnology for crop improvement.

## Overview of Gene Editing Tools

**Figure 1.**
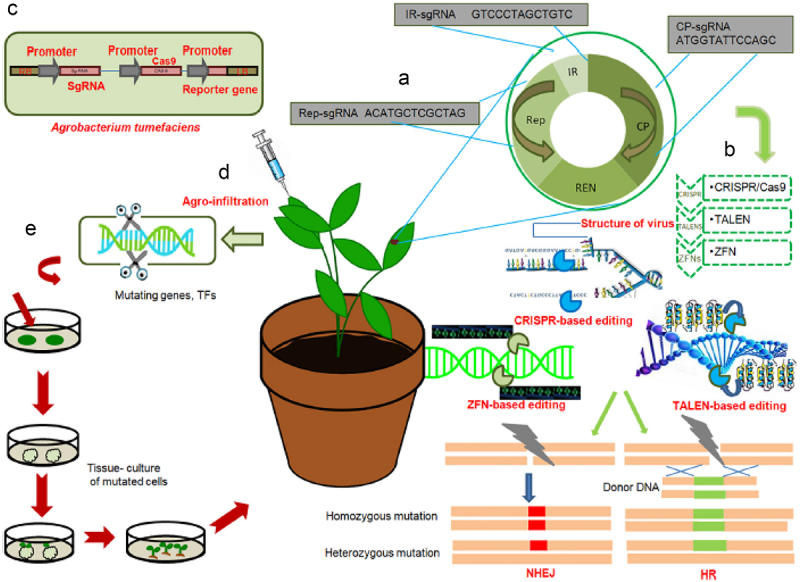
The general workflow for gene editing technologies to engineer disease resistance in crops, (a) general structure of the viral genome; target single-strand RNAs (sgRNAs) containing putative sequences are displayed in red for the replication-associated protein (Rep), intergenic region (IR), and viral capsid protein (CP) regions of the viral genome. CRISPR/Cas9 can be used to execute a multiplex genome editing technique based on multiplex sgRNA targeting IR, CP, and Rep of several viruses. (b) A diagram illustrating three methods for modifying the genome that give plants immunity against genomic alterations at specific sites. Bacterial or viral genomes undergo induced mutagenesis, which makes them ineffective. (c) Agrobacterium tumefaciens T-DNA producing reporter gene (GFP) under CaMV promoter, Cas9 protein under CaMV promoter, and sgRNA under CaMV promoter. (d) Plant cell agroinfiltration, which involves injecting an Agrobacterium bearing an engineered virus into a Cas9-expressing plant to express the target virus’s sgRNA. (e) Editing the genome of genes or transcription factors to adversely regulate a plant’s resistance to bacterial, viral, or fungal infections by deleting certain base pairs, followed by the cultivation of a resistant plant by tissue culture methods.^52^

Genome editing encompasses a collection of techniques rather than a single technology or approach that allow for more precise genome alteration than in earlier versions of genetic engineering.^48^ This technology presents promising solutions for mitigating the negative impacts of climate change on food security. Genome editing tools include Zinc-Finger Nucleases (ZNFs), Transcription Activator-Like Effector Nucleases (TALENs), and Clustered Regularly Interspaced Short Palindromic Repeats (CRISPR/Cas9).^49–51^ Their goal is to add, delete, or alter DNA nucleotides in order to influence them (Figure 1).^48^ Leveraging on ZNFs, TALENs, and CRISPR/Cas9, scientists have the capacity to engineer crops that exhibit increased resilience to evolving environmental conditions, including heat stress, drought, and diseases. These innovative techniques facilitate precise modifications in the genetic composition of plants, leading to the generation of new types that are resilient to heat, resistant to diseases, and high-yielding. The fast advancement of genome editing methods enables the accelerated generation of climate-resilient crop varieties, which is required to maintain global food security in the face of climate change problems.

NHEJ; non-homologous end joining, HR; homology repair.

## Zinc Finger Nucleases (ZFNs)

**Figure 2.**
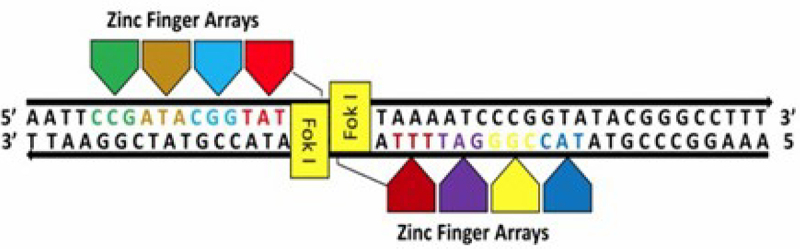
Diagram illustrating how zinc finger nucleases (ZFNs) are used to alter the plant genome: target specificity is achieved by zinc finger arrays connected to FoKI endonuclease.^65^

Plant genome editing was initially accomplished in 2005 with the use of ZFNs, the earliest generation of genome editing instruments.^53^ ZFNs are synthetic nucleases that are produced by joining a nonspecific DNA cleavage domain that is sourced from the type II restriction endonuclease FokI with an artificial, sequence-specific zinc finger DNA binding domain FokI.^54^ During the initial phases of genome editing, researchers had to create many zinc-finger nucleases (ZFNs) to induce the necessary double-stranded breaks (DSBs) at precise locations in the DNA (Figure 2).^55,56^ This nuclease system required specialized knowledge to produce synthetic proteins with DNA-binding domains that could each bind a different sequence and be coupled to a nonspecific nuclease for target cleavage.^56^

The resulting zinc-finger domain’s functional specialization includes a wide variety of *Cys*_*2*_*His*_*2*_ (C_2_H_2_) zinc fingers (ZFs) – which functions as trans-regulators of gene expression, which emerge from highly stable contacts between their zinc-finger domains and homologous DNA sequences. A single *Cys*_*2*_*His*_*2*_ (C_2_H_2_) zinc finger normally consists of about 30 amino acids, which combine to create a α-helix and two opposing β sheets.^57^ The *Cys*_*2*_*His*_*2*_ zinc finger motif is the predominant type of DNA-binding motif observed in eukaryotic transcription factors.^58^ It is a flexible DNA recognition domain.^59^ Each zinc-finger unit specifically identifies three base pairs (bp) of DNA and creates base-specific interactions by interacting with the main groove of DNA through its α-helix residues.^60^ DNA is cleaved by the FokI type II restriction endonuclease and could be utilized as a dimer to accurately focus particular sections in the genome, enabling effective gene editing.^61^ After ZFNs successfully cleave DNA in eukaryotic cells, DSBs are created at a specified location in the genome, which causes the required changes in subsequent non-homologous end joining (NHEJ) or homology-directed repair (HDR) systems.^54^ Three critical aspects determine the sequence of interest identification and accuracy of ZFNs: (a) each finger’s amino acid sequence; (b) the number of fingers; and (c) the fingers’ association with the nuclease domain.^62^ Early investigations utilized single zinc finger nucleases (ZFNs) consisting of 3–6 fingers to interact with targets that were 9–18 nucleotides long enabling ZFN dimers to accurately identify and attach to 18–36 base pairs of DNA at each cleavage site.^63^ The use of an 18 bp DNA sequence can achieve specificity within 68 billion bp of DNA, allowing to aim at particular regions in the human genome for the first time. In a more modern concept, architectural diversification was utilized to increase ZFNs’ targeting accuracy.^64^ This work created a new connector alternative for bridging finger-finger and finger-FokI cleavage domain junctions, increasing the number of ZFN combinations that may be used to target cleavage to a specific DNA base by a factor of 64.

## Transcription Activator-Like Effector Nucleases (TALENs)

**Figure 3.**
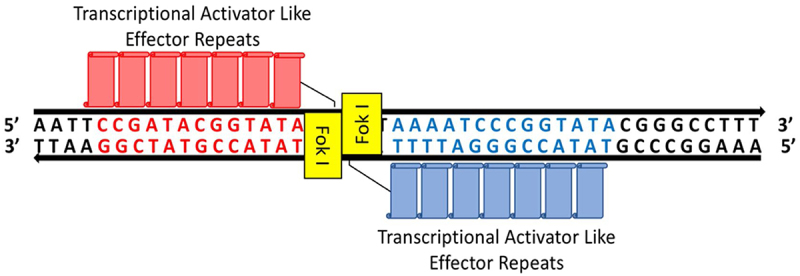
Diagram illustrating how the transcription activator-like effector nucleases (TALENs) are used to modify the plant genome. There are 13–28 transcriptional activator like effector repeats (TALE repeats) in the DNA binding domain of TALENs. Sticky DSBs are produced by TALENs.^65^

Transcription activator-like effector nucleases (TALENs), the second frequently employed gene-editing technique, is a comparatively recent gene-editing technology. The word “TALEN” originally referred to a group of eukaryotic zinc finger proteins with cleavage domains that infect rice and are produced by the pathogenic bacterium Xanthomonas. Located in the center of the protein, TALEN is a highly repetitive and conserved region made up of tandem repeats, the majority of which have 33 or 34 amino acid segments with polymorphic 12 and 13 repeat variable diresidues (RVDs).^66–69^ In addition, researchers have studied the DNA of bacteria other than *Xanthomonas* and found that *Ralstonia solanacearum* (Ralstonia) has Ralstonia TALE-like proteins (RTLs).^70^ The way these RVDs may be utilized to create TALEN-based engineering systems was revealed through deciphering of the code of RTL binding to DNA.^71^ However, TALEN-based genetic engineering is demanding in terms of both labor and resources since it necessitates the creation of a specific protein for any target and simultaneously binding two TAL monomers to the DNA strands.^50^ The novel TALENs are currently regularly utilized for genetically modifying zebrafish, chickens, frogs, rats, mammalian cells, and dengue vector (Figure 3).^72,73^

## Clustered Regularly Interspaced Short Palindromic Repeats/crispr-Associated Protein (Crispr/cas)

Clustered regular interspaced short palindromic repeats/CRISPR-associated protein (CRISPR/Cas) is a newly identified and potent gene editing tool formed via a bacterial receptive immunity mechanism.^74^ The structure includes a group of CRISPR-associated (Cas) genes that produce Cas proteins with an endonuclease function, as well as CRISPR repeat-spacer series that can be synthesized into CRISPR RNA (crRNA) and trans-activating CRISPR RNA (tracrRNA).^75^ Due to its straightforward design, affordability, high efficiency, consistency, and quick cycle capabilities, CRISPR/Cas9 systems are often utilized for genome editing.^76,77^

**Figure 4.**
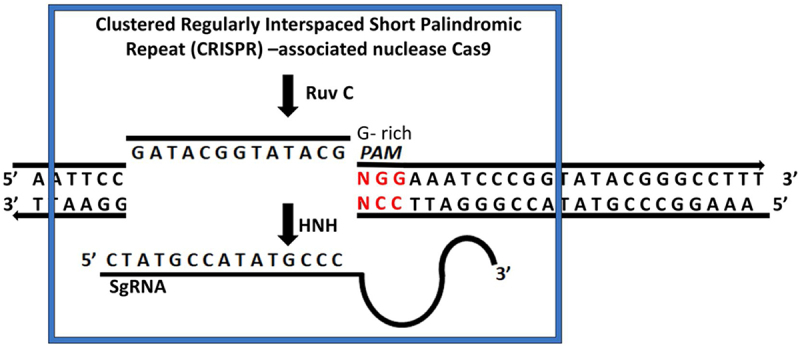
Diagram illustrating how the CRISPR/Cas9 is utilized to alter the plant genome. Leveraging on HNH and RuvC domains, the Cas9 nuclease aims at the complementary and non-complementary DNA to create a blunt end DSB.^65^

There are two classes of the CRISPR/Cas9 systems (Class 1 and Class 2), six kinds (I to VI), and several subtypes. While Class 2 systems (Type II, V, and VI) have a single effector protein, Class 1 systems (Type I, III, and IV) contain numerous Cas protein effector complexes.^78–81^ One of the greatest and most widely utilized CRISPR-Cas9 systems is Type II, or SpCas9, which is specially developed from *Streptococcus pyogenes*.^82–84^ A single-guide RNA (sgRNA) and an RNA-guided Cas9 endonuclease are the two main components of the CRISPR-Cas9 pathway.^85^ The Cas9 protein has two nuclease fields, known as HNH and RuvC, and each cleaves one strand of the intended double-stranded DNA (Figure 4).^86^ A single-guide RNA (sgRNA) is an integration of crRNA and tracrRNA.^87^ A Cas9 ribonucleoprotein (RNP), which is composed of the Cas9 nuclease and sgRNA, has the ability to bind to and cleave a particular DNA target.^78^ Likewise, a protospacer adjacent motif (PAM) sequence must exist for the Cas9 protein to attach to the targeted DNA.^85^

The RNA-guided system, or CRISPR/Cas9, is more suitable for implementation and provides some noteworthy improvements as compared to earlier gene editing techniques.^88^ Multiple loci may be edited simultaneously by using CRISPR/Cas9, making it a more cost-effective, faster, and more widely available methodology compared to previous genome editing methods.^62^ Table 1 provides a summary of the different genome editing techniques.

**Table 1.** Comparison of the genome editing tools ZFNs, TALENs, and CRISPR/Cas9.

Functions	ZFN	TALEN	CRISPR/Cas9	Reference
Components	Zn finger domains Nonspecific FokI nuclease domain	TALE DNA-binding domains Nonspecific FokI nuclease domain	crRNA, Cas9 proteins	89
Structural Protein	Dimeric protein	Dimeric protein	Monomeric protein	90
Catalytic domain	Restriction endonuclease FokI	Restriction endonuclease FokI	RUVC and HNH	78
Length of target sequence (bp)	24–36	24-59	20–22	90,91
gRNA production	Not applicable	Not applicable	Easy to produce	92,93
Mode of action	Double-strand breaks in target DNA	Double-strand breaks in target DNA	Double-strand breaks or single-strand nicks in target DNA	93,94
Target recognition efficiency	High	High	High	89,90
Mutation rate	High	Middle	Low	90
Multiplex targeting	Difficult	Difficult	Possible	93,94
Off-target effects	Low off-target effect	Shows least off-target activities	Low off-target effect	95
Cost of development	High	Higher	Low	96

## Genome Editing and Other Technologies of Crop Improvement

Plant breeders have been leveraging conventional approaches to improve crops. These crop improvement techniques include selective breeding, hybridization, mutation breeding, and tissue culture, among others.^97^ Selective breeding involves the deliberate selection and mating of organisms with desirable traits.^98^ This method has been employed for millennia to enhance crop characteristics. By repeatedly selecting and crossing individuals with preferred attributes, breeders can gradually improve crop yield among other desirable traits.^99^ However, this process is time-consuming and depends on the availability of genetic variation in the existing gene pool, limiting the potential for novel trait introduction.^100^ To overcome the limitations of selective breeding, hybridization was introduced as an alternative approach to introduce new genetic variation into crop plants^101^

Hybridization combines the genetic material of different plant varieties or species to create new cultivars.^102^ This technique can introduce desirable traits from one species into another, expanding the genetic diversity available for crop improvement.^103^ However, hybridization often leads to hybrid vigor, where the first-generation offspring exhibit superior performance but subsequent generations may experience reduced vigor and uniformity.^104^ Additionally, it can be challenging to combine desired traits while maintaining agronomical important characteristics.^104^ To overcome the unpredictability associated with hybridization, inducing mutations became another approach to generate genetic diversity for crop improvement.^105^

Mutation breeding involves inducing random genetic changes in plants to create new variants with desirable traits.^106^ This can be achieved through physical or chemical mutagenesis. Although this approach has produced valuable crop varieties, it is inefficient as the vast majority of induced mutations are deleterious.^107^ Identifying beneficial mutations from a large pool of random variants is time-consuming and resource-intensive.^108^

The limitations inherent in traditional breeding methods, such as their reliance on existing genetic variation and the unpredictable nature of induced mutations, have necessitated the development of more precise and efficient crop improvement technologies. Consequently, the advent of biotechnology such as Marker Assisted Selection (MAS), Transgenic Crops (TrC), and Tissue Culture (TC) has brought about an entirely novel phase of crop enhancement.^109^ MAS uses molecular markers to select individuals that harbor desired traits more efficiently.^110^ By correlating specific DNA sequences with phenotypic traits, breeders can select superior individuals for breeding purposes without relying solely on phenotypic evaluation. This technique accelerates the breeding process and increases the accuracy of selection. However, MAS is dependent on the availability of reliable molecular markers for target traits, which may not be available for all desired characteristics.^111^

Transgenic crops involve the introduction of foreign genes into a plant’s genome to confer entirely distinct characteristics.^112^ This is achieved through methods like Agrobacterium-mediated transformation or gene gun delivery.^113^ Transgenic crops have the potential to address various agricultural challenges, including increased yield, improved nutritional content, and enhanced pest and disease resistance.^114^ However, the development and commercialization of transgenic crops are often met with regulatory hurdles and public concerns regarding potential environmental and health risks.^115^ To complement transgenic technologies and address issues related to clonal propagation and disease eradication, tissue culture emerged as a valuable method in crop enhancement.

Tissue culture involves the growth of plant cells, tissues, or organs under sterile conditions.^116^ This technique enables the propagation of elite plant genotypes, clonal propagation, and the generation of healthy plants.^117^ Furthermore, it is essential for crop improvement programs as it offers a dependable and efficient method to produce many identical plants.^118^ It is also useful for the regeneration of transformed plants. However, it can be technically challenging and expensive, limiting its widespread application in developing countries.^119^

The limitations of these biotechnology techniques, such as the reliance on available genetic variation, the challenges associated with transgene integration, and the technical complexities of tissue culture, have prompted the search for more precise and efficient crop improvement tools.^120^ In order to get beyond these challenges, genome editing technologies such as CRISPR-Cas9 have become a revolutionary method that is revolutionizing the field of plant sciences. Genome editing, spearheaded by CRISPR-Cas9, has transformed crop improvement through precise and efficient genetic manipulation.^121^ This technology involves guiding the Cas9 enzyme to a precise DNA sequence using a complementary RNA molecule, where it induces a double-stranded break. The cell’s repair mechanisms can then be harnessed to introduce desired genetic modifications.^122^ This burgeoning technology holds enormous promise to enhance crop yield, nutritional content, and resilience to biotic and abiotic stresses is immense.^123^ Plant genome editing offers the ability to reduce breeding program duration, which contrasts sharply with traditional breeding methods, boosts consumer acceptance, and has reduced regulatory limitations relative to transgenic breeding techniques.^124^ Genome editing is a game changer for crop improvement in SSA and will be more adoptable by the populace compared to those that were genetically engineered for several reasons. Unlike genetic engineering, which can introduce unintended changes in the plant genome, genome editing is more precise as it permits targeted specific edits to existing genes, which reduces the risk of off-targets. This makes it a more reliable tool for crop improvement. Furthermore, the technology is faster and cost-effective, and highly efficient in accelerating breeding programs, especially with regard to mitigating the effects of climate change currently ravaging some countries in the region. To complement genome editing, other crop improvement strategies, including conventional breeding such as selective breeding, hybridization, and mutational breeding together with biotechnology techniques, continue to play crucial roles in agricultural advancement.^125^

## Applications of Gene Editing in Mitigating Effect of Climate Change

Numerous genome editing techniques target certain locations in the genome and have the ability to increase, decrease, or suppress the activity of a particular gene or genes.^126^ The most popular technique for accurate genome editing is CRISPR/Cas9 because of its remarkable efficiency, cost-effectiveness, and easy implementation.^127–129^ The underlining principle of this technology is that it works like a molecular scissor that makes it possible to edit a precise location in an organism’s DNA. CRISPR/Cas9 tool makes it possible to locate particular genes coding for traits and either to on (activate) or off (silence) the gene based on the sort after effect.^130^ The edited DNA is restored with time and the new traits are passed on to the progenies.^131^ The pursuit of improving crop production persists through the utilization of genome editing as a promising method. Currently, the majority of worldwide research funding for crop enhancements is focused on rice, maize, wheat, banana, and cassava. These crops serve as the source of staple food in SSA. In many African countries, researchers are utilizing genome editing to develop new, resilient varieties of staple crops tailored for smallholder farmers. This technology offers the potential to produce crops that can withstand the challenges of climate change. By integrating genome-edited crops with traditional farming practices, agriculture in SSA could be significantly transformed, addressing the urgent problems faced by farmers. Success results have been recorded in some staple crops across SSA:

## Rice

Rice is positioned as the main diet supply for more than half of the globe’s population, serving a significant role in broad food security.^132,133^ As faced by all food crops, rice production is confronted by two main abiotic stresses, namely, salinity and drought, which calls for research studies on ways to solve them by leveraging the genome editing technique to create resilient cultivars. Zhang et al.,^134^ utilized CRISPR/Cas9 to knock-out *ORYZA SATIVA RESPONSE REGULATOR 22 (OsRR22)* gene which is linked to salt susceptibility in rice. The edited rice performed well in high salinity environments without any recorded reduction in grain yield and plant biomass. In addition, with the advent of gene editing, rice varieties tolerant to drought and high temperatures are now available. Yin et al.,^135^ focused on positive regulator of stomata density. The research found that rice lines that were genetically modified to have fewer stomata had a higher yield when exposed to severe dry conditions.

Yue et al.,^136^ reported that inhibiting or knocking out *OsmiR535* which controls the activity of abiotic stress-responsive significantly improved rice’s tolerance to salt salinity. This enhancement was evident through increased lateral root development, primary root length, and shoot length. Specifically, the shoot length in the *OsmiR535* mutant and STTM535 transgenic plants was 86.8% and 66.72% greater than that of wild-type plants, respectively. Additionally, the primary root length was 35.8% and 31.3% longer in these plants compared to the wild type. Overall, the inhibition or knockout of *OsmiR535* effectively bolstered salinity tolerance by promoting better root development.

Bacterial leaf blight is a highly destructive rice disease globally.^137,138^ Zeng et al.,^139^ used CRISPR/Cas9 to induce mutation on *ORYZA SATIVA SUGARS WILL EVENTUALLY BE EXPORTED TRANSPORTER 14* (*OsSWEET14*) gene, which is involved in bacterial blight resistance and encodes sugar transporter. Gene edited rice lines expressed resistance to both Asian and African *Xanthomonas* strain without yield penalties. Zhou et al.,^140^ edited *BROAD-SPECTRUM RESISTANCE KITAAKE-1 (bsr-k1)* gene in rice by employing CRISPR tool. The edited bsr-k1 gene enhanced the defense system of disease resistance in rice against both leaf blast and bacterial leaf blight. The edited rice lines exhibited an increment of 50% yield when exposed to both leaf blast and bacterial leaf blight on the field.

A further investigation showed that the introduction of modifications to the *OsRR*22 gene, which encodes the reaction regulator of the type-B variety, resulted in rice plants exhibiting a significant increase in their ability to withstand high levels of salt.^134^ Furthermore, the deployment of CRISPR/Cas9 led to the production of *Oryza sativa pseudo-response regulator* (*ospqt*3) mutants, which exhibited a significant enhancement in salt tolerance in rice.^141^

Research explorations have resulted in the production of early maturing rice varieties. CRISPR/Cas was applied on Heading date 2 (Hd2), 4 and 5 genes coding for flowering in rice. This has led to the release of rice plants that flower early relative to the landraces.^142^

## Wheat

Even though Wheat (*Triticum aestivum* L.) occupied 17% of the total arable land, Africa spends 12.74 billion USD annually for wheat imports, which is 15% of all food import.^209^ Water shortage, salinity, and excessive temperature are factors contributing to reduced wheat output.^143^ The CRISPR/Cas9 gene editing method, which is currently being developed, provides a very reliable approach in wheat improvement.^144^ The *SAL1* gene is crucial for drought tolerance because of its transcripts for enzymes with diverse activities, including the ability to respond to oxidative stress.^145^ The absence of active *SAL1* enhanced drought tolerance in tobacco and Arabidopsis.^71,146^ The *SAL1* gene, which acts as a negative modulator of resilience to drought in wheat, was modified using a multiplex CRISPR/Cas9 test. This modification enhances drought tolerance throughout the seedling period of growth.^147,148^ Powdery mildew is a destructive disease that impairs the best possible outcome of wheat production.^72^ The application of genome editing technology using CRISPR/Cas9 and TALENs has effectively edited the mildew resistance site in the wheat genome, leading toward success.^149^ The modified wheat varieties displayed immunity to the powdery mildew disease, showing no vulnerability to the illness compared to the original variety.

## Maize

Maize (*Zea mays* L.) is a vital staple food crop in SSA, providing both nutrition and support to the livelihoods of small-scale farmers.^150^ The disparity between the expected and the actual grain production is attributed to both biotic and abiotic stresses.^151^ A major abiotic stress in areas where maize is cultivated is drought, resulting from low and unpredictable rainfall.^152^ Extreme droughts affect 25% of the maize crop, potentially resulting in harvest losses of up to 50%.^153^ CRISPR/Cas9 technology was used to modify the *AUXIN-REGULATED GENE INVOLVED IN ORGAN SIZE* (ARGOS8) gene in maize. This gene improves drought tolerance and governs the plant’s response to ethylene.^154^ This modification increased adaptation to drought in the maize plants.

In collaboration with Corteva Agriscience and the Kenya Agricultural and Livestock Research Organization (KALRO), CIMMYT scientists are pioneering the development of maize lines resistant to Maize Lethal Necrosis (MLN) using CRISPR-Cas9 technology. Maize lethal necrosis (MLN) was first identified in Kenya in 2011, it led to a 22% reduction in maize yields by 2013, resulting in $180 million in lost production and forcing many smallholder farmers to abandon maize cultivation.^155^ To combat this, researchers selected four MLN-susceptible lines, which are the parent lines of two widely popular, stress-tolerant but MLN-vulnerable hybrids developed before 2011 in Kenya and Uganda.^150^ These lines are being edited to confer MLN resistance, ensuring that the resulting hybrids retain all farmer-preferred traits, including stress tolerance, while gaining the crucial advantage of MLN resistance. By 2025, pending regulatory approval, commercial seeds of these gene-edited, MLN-resistant maize hybrids will be available to 20,000 smallholder farmers, covering approximately 40,000 hectares.

## Cassava

Cassava (*Manihot esculenta* Crantz) is a significant crop primarily cultivated for its nutritious storage roots and to a lesser extent for its foliage in tropical and subtropical areas.^156,157^ Two viral diseases, cassava mosaic disease (CMD) and cassava brown streak disease (CBSD), along with a bacterial disease known as cassava bacterial blight (CBB), are the main obstacles to cassava farming in Africa.^158^ The CMD is very destructive and can lead to crop damage ranging from 20% to 100%.^159^ CBSD is a severe viral infection in cassava plants brought about by two species of positive sense RNA virus from the family Potyviridae, specifically the genus Ipomovirus.^160^ The primary mode of transmission is by the whitefly (*Bemisia tabaci*), which acts as a vector for the virus.^161^ The projected yearly losses caused by CBSD in East Africa alone amount to over $175 million.^162^ No evidence of a cassava cultivar with full immunity to CBSD has been seen among the cultivars used by farmers.^163^ Studies have shown that CBB can lead to a complete decline in productivity, reaching up to 100%, in environments with high levels of humidity and wetness, which create ideal circumstances for maximal extent.^164^ *NUCLEOTIDE-BINDING LEUCINE-RICH REPEAT* (*NBS – LRR*), known as recessive resistance genes, offer a robust defense against certain pathogens.^165,166^ Gomez et al.^167^ found that the presence of the potyvirus as a recessive gene, when combined with eif4E proteins known as novel cap-binding protein n CBP-1 and n CBP-2, might increase the occurrence of CBSD in cassava. The modification of cassava genes using CRISPR/Cas9 technology enhances the plant’s resistance to viral infections, as demonstrated by a reduction in the incidence of Turnip Mosaic Potyvirus (TuMV) attacks on *Arabidopsis thaliana* and cucumber in studies conducted by Chandrasekaran et al.^168^ and Duprat et al.^169^

In Uganda, Odipio et al.^170^ provided proof of concept by editing local cassava varieties with the aim of altering flowering time. Validating this concept could facilitate the induction of fertile flowers and seeds in cassava, leading to substantial advancements in cassava improvement. Cassava leaves and roots contain toxic amounts of cyanogenic glycoside linamarin, which can cause cyanide poisoning upon conversion to cyanide in the body. In Kenya, Juma et al.^171^ successfully employed CRISPR-Cas9 to lower cyanide levels in cassava by targeting mutagenesis of the *MeCYP79D1* gene in exon 3 through CRISPR/Cas9 and *Agrobacterium*-mediated transformation. *MeCYP79D1* gene encodes the valine monooxygenase I enzyme involved in biosynthesis of cyanogen in cassava. They achieved a reduction in linamarin and cyanide levels in the leaves of MeCYP79D1 transgenic lines by seven-fold. These findings demonstrate that CRISPR/Cas9-mediated mutagenesis can be a game-changing strategy for developing cassava with reduced cyanide content, paving the way for the creation of safer cassava cultivars for smallholder farmers in SSA.

## Banana

Banana (*Musa* spp) has been established as a primary food crop in tropical and subtropical regions of Africa, with a yearly yield of about 155 million tons.^172^ Nevertheless, the cultivation of bananas in the SSA region is significantly hindered by a severe disease called *Banana Xanthomonas Wilt* (BXW), which is triggered by a bacterium called *Xanthomonas campestris pv. musacearum* (Xcm).^173^ This disease has resulted in substantial economic losses, between 2 and 8 billion US dollars in the affected countries.^210^ To avoid BXW, Tripathi et al.^174^ developed a CRISPR/Cas9-edited banana by creating changes in *MUSA DOWNY MILDEW RESISTANCE 6* (*MusaDMR6*) orthologues. It encodes the enzyme 2 oxoglutarate Fe (II)-dependent oxygenase (2OGO), which is up-regulated by pathogens when they are infected.^175^ An orthologue of *MusaDMR6* gene known as the Ma04_p20880.1 was chosen as a possible option for enhancing immunity to diseases in bananas. When compared to non-infected plants, the Sukali Ndiizi cultivar’s qRT-PCR investigation on the *in vitro* plantlets revealed a substantial increase in the expression of the Ma04_p20880.1 gene. When compared to non-infected plants, the Sukali Ndiizi cultivar’s qRT-PCR investigation on the *in vitro* plantlets showed a substantial increase in the expression of the Ma04_p20880.1 gene. This investigation demonstrated that the Ma04_p20880.1 performs a comparable role to that of *AtDMR6*, which plays a role of balancing defense response. The up-regulation of the Ma04_p20880.1 gene was not found in the BXW-resistant genotype of *Musa balbisiana*, indicating that its activity was suitable for an association between the plant and the pathogen. The Ma04_p20880.1 gene from *Musa acuminata* (AA genome) and the corresponding gene *ITC1587_Bchr1_T00081* from *Musa balbisiana* (BB genome) were employed to modify Sukali Ndiizi (AAB genome) cultivar, which is susceptible to BXW, using *agrobacterium*-mediated transformation.^176^ The findings demonstrated that 25 out of 30 genetically altered plants showed enhanced immunity to BXW, whereas unaltered plants exhibited signs such as browning and wilting significantly sooner (after 14.7 days) under a regulated *in vitro* setting. Subsequent experimentation conducted in a greenhouse revealed that 52% (12 out of 23) of the subjects had enhanced resistance to BXW. In order to verify the stability of the mutation, new plants were cultivated from the changed plant, specifically by the process of micropropagation. It was seen that the mutations remained present and exhibited stability in these newly produced plantlets.

Another biotic stress that constrains banana production is the Endogenous Banana Streak Virus (eBSV), found within the banana genome, which poses a significant challenge for banana breeding. These eBSV sequences originates from *Musa balbisiana*.^177^ Under stress conditions, eBSV in banana plants can recombine to form a functional episomal viral genome, leading to the production of infectious viral particles and the manifestation of disease symptoms. *In vitro* propagation and conventional breeding through hybridization may also activate this virus, further complicating breeding efforts. Consequently, Banana Streak Virus (BSV) significantly limits the use of the diploid progenitor *Musa balbisiana* or its B genome derivatives as parent plants for introducing desirable agronomic traits.^178^ To address this challenge, scientists at the International Institute of Tropical Agriculture, Kenya, have leveraged on CRISPR-Cas9 technology to inactivate the eBSV sequences in the genome of the Gonja Manjaya plantain variety.^211^ Their work demonstrated that CRISPR/Cas9-mediated targeted mutagenesis can permanently deactivate endogenous eBSV, offering a promising approach for the inactivation of other endogenous viral genomes. This study paves the way for editing banana germplasm with B genome(s), making them viable parental lines in breeding programs. The significance of this groundbreaking study for Africa is immense, given the vital role that banana plays as a staple food in SSA. This advancement allows for the improvement of B genome germplasm and its incorporation into breeding programs to develop hybrids that are better adapted to SSA agricultural environments.

Delay Ripening Banana is a climacteric fruit that becomes soft in texture as it ripens. Typically, bananas ripen rapidly and begin to deteriorate within a week. Banana fruit can have its shelf life, delivery, and selling potential increased by delaying ripening, all while reducing postharvest damage. Using CRISPR/Cas9 technology, the banana genome was edited to target the *AMINOCYCLOPROPANE-1-CARBOXYLASE OXIDASE 1* gene (*MaACO1*) which promotes ethylene biosynthesis and accelerates fruit ripening. This resulted in a delay in the ripening process, leading to an extended shelf life of the fruit.^179^ The modified banana fruits exhibited decreased ethylene production and prolonged durability in their natural state. This promising outcome suggests the potential to develop BXW-resistant banana varieties, which could enhance production and mitigate the effects of bacterial diseases on smallholder farmers in East Africa. Table 2 shows the various genome editing tools used in crop improvement.

**Table 2.** Genome editing tools in crop improvement and their functions.

Genome editing tool	Crop	Use	References
ZFNs	Soybean	Herbicide transference	180
	Maize	Enhanced resistance to herbicides	181
	Rice	Identification of safe harbor loci for herbicide resistance	182
TALENs	Rice	Enhanced fragrance	183
	Wheat	Improved resistance to Powdery mildew	149
	Potato	Enhanced resistance to herbicide	184
	Sugarcane	Reduced lignin content and enhanced biofuel production	185
CRISPR/Cas9	Banana	Increase yield	186
	Cassava	Quality improvement	170
	Wheat	Generate plantlets from somatic embryo	187
	Sorghum	Increase grain protein digestibility and lysine content	188
	Rice	Improvement of grain weight and early maturity of rice varieties	142,189

## Limitations of Gene Editing Tools

Despite the proven efficacy of ZFNs in modifying genomes of both plants and animals, their practical use presents numerous obstacles. The major limitations in applying ZFNs encompass low efficacy, high cost of protein domain construction, a restricted array of targets, more context-dependent effects among repeat units, low targeting precision, and frequent off-target effects.^190–192^ The subsequent production of nucleases specific to certain sequences, such as CRISPR/Cas9 and transcription activator-like effector nucleases (TALENs), is characterized by their regular spacing and clustering, providing a simpler design of construct and improved efficiency compared to ZFNs.^193^ TALENs are more readily created and produced although they suffer from off-target binding.^194^ In addition, difficulties in protein engineering have constrained wide adoption of TALENs for genome editing.^191^ Both ZFNs and TALENs rely on the protein engineering of DNA binding domains for the identification and modification of particular DNA sequences. Limitations in the application of these genome editing may arise from the potential inefficacy, tedious nature, and high cost. The above challenges have found a resolution in the emergence of CRISPR/Cas9 system.^195^ The CRISPR/Cas9 is preferred over ZFNs and TALENs due to its cost-effectiveness and has brought about a significant transformation within the biotechnology field^196,197^.

Despite CRISPR/Cas9 system being a game-changer, it faces challenges that limit its effectiveness. These challenges include off-target effects, low efficiency, and poor delivery mechanisms.^130^ Previous limitations in CRISPR technology have been significantly ameliorated by new technical advancements in the technology. A way to reduce accidental changes in Cas9 systems is by combining Cas9 with ZFNs or TALENs, which can target almost any part of the genome more accurately.^198^ Cas9 mutants can lessen unwanted DNA interactions by making the bond between Cas9 and the target DNA strand or the non-target DNA strand weaker while still cutting the intended DNA precisely.^199,200^ Furthermore, new versions of CRISPR/Cas9 are better at targeting specific parts of the DNA sequence by recognizing different protospacer adjacent motifs (PAMs).^201^ Moreover, other methods have been created to reduce these accidental changes, such as identifying off-target effects, using cytosine and adenine base editors, prime editing, and shortened gRNAs that better match the target sequences.^202^

Another bottleneck that hinders the effectiveness and specificity of CRISPR/Cas9 technology is the delivery methods. CRISPR is not naturally found in plants, and as a result, CRISPR/Cas9 proteins must be introduced into plant cells, which takes a lot of time.^203^ Hitherto, the CRISPR/dCas9 leverages on Polyethylene glycol (PEG)-mediated transformation, Agrobacterium-mediated transformation, biolistic transformation, and particle bombardment as the delivery systems. However, a new method that directly transfers Cas9 constructs into the plant’s apical meristem, avoiding tissue culture, is helping to overcome this problem.^204^

In addition to these technical limitations of genome editing, SSA faces several challenges in the area of capacity to conduct biotechnology research and the lack of regulation for genome editing in many countries in the region. Public research organizations still lack the laboratory facilities and funding necessary to implement the latest genome editing methods. Furthermore, biosafety regulations remain excessively stringent throughout the continent, impeding innovation.^205^ The region's underdeveloped regulatory systems make it difficult for biotechnology to significantly improve the livelihoods of smallholder farmers.^206^ Gene editing has enormous possibilities for crop enhancement in SSA; however, these challenges must be overcome to completely utilize the benefits of gene editing in African agriculture.

## Regulation of Genome Edited Crops

The regulatory landscape for plant genome editing technologies in Africa is diverse. There are disparities in the definition of Genome-Edited (GEd) Crops and Genetically modified Organisms (GMOs).^207^ In terms of regulation, unlike genetic engineering which undergoes stringent regulations, genome edited crops do not all introduce foreign genes and therefore overcome the hurdles of such stringent regulations which enhance its adoption. Regulation differs in the types of SDN. For instance, SDN-1 is generally regulated as GMOs, with varying levels of strictness across different jurisdictions, with the EU having stricter regulations compared to the US. SDN-2 and SDN-3 may be regulated differently, as they do not make double-stranded breaks or introduce foreign DNA.^208^ Regulations of genome edited crops differ across countries. Nevertheless, countries like the US, Canada, and UK adopt the same rule. The regulatory framework in these countries is product-based, emphasizing the qualities of the finished product over the method of production. In general, the end result of gene-edited plants is thought to be identical to a product produced without gene editing if it does not contain any foreign DNA. The extra rules that products made using other kinds of biotechnology procedures must abide by would not apply to the plant if it could have been developed by conventional breeding.^172^ Recently, the United Kingdom passed a law that mandates that gene-edited plants must be developed, released, and marketed under a distinct regulatory framework than plants created using other biotechnology procedures, such as genetic engineering.^212^ In the case of Africa, genome editing is at its nascent stage with only two countries, Nigeria and Kenya having regulatory frameworks for the technology on a case-by-case basis. This is consistent with Agenda 2063 of the African Union, which aims to use genome editing to increase resilience of crops and agricultural output.^34,126^ Nigeria took the lead in 2020 with guidelines on genome editing given by the National Biosafety Management Agency, which approves gene edited crops on a case-by-case basis, provided they do not contain foreign genetic material. On the other hand, Kenya’s National Biosafety Authority gave their guidelines in 2022 which exempted genome edited crops alongside goods from the Biosafety Act.^34,126^ They are at the forefront on the continent when it comes to creating official standards for integrating GEd and other innovations into legal structures.^207^

South Africa regulates GEd crops as GMOs.^213^ Despite having been at the forefront of regulating GMOs for commercial purposes on the continent, South Africa has been sluggish to adapt its legal regulations to GEd requirements.^207^ In some Eastern African countries, specifically Ethiopia and Uganda, discussions on developing guidelines specific to GEd are still ongoing. Laws and regulations associated with GEd have been published in Burkina Faso, Ghana, Kenya, Nigeria, and Malawi, excluding from GMO regulation GEd products devoid of foreign genetic material.^213^

There are variations in GEd regulation even among members of a common trading bloc, which makes cross-border technology up scaling difficult. Kenya, Uganda, and Tanzania are in the same trading block and have common agricultural constraints, yet they do not have the same view on GEd.^213^ Kenya has a robust genetically engineered product regulatory system that supports cutting-edge technology to stave off climate change and preserve food security.^214^ Conversely, Uganda is at the nascent stage of crafting GEd-specific directives, despite having the necessary biotechnology research capabilities and a well-qualified scientific community.^215^ However, Tanzania, another member of the East African Community (EAC), still lacks explicit regulations for GEd products.^214^ As global trends lean toward more lenient regulation of GEd products relative to GMOs, African countries ought to establish risk-appropriate, harmonized legislation to effectively govern GEd technologies.^216^

## Genetic Engineering Technologies Adoption Challenges in SSA

The legislative frameworks adopted by African nations create a complex environment that influences both domestic crop production and international partnerships in the deployment of genetically engineered crops. These approaches vary widely, from strict restrictions to the deliberate embrace of such technologies;^214,217^ Sadikiel.^218^ The adoption of genetically modified crops in African agriculture has been hampered by ethical concerns, slowing the spread of this rapidly advancing technology across much of Sub-Saharan Africa.^219–221^ Additionally, diverse cultural frameworks and socioeconomic conditions have shaped the broad range of opinions and perspectives held by African populations regarding genetically altered crops;^222^ Sadikiel.^218^

Another bottleneck that hampers the adoption of genetic engineering technologies in Africa is the lack of political will, external influence, and a general lack of knowledge and awareness about the scientific foundations of these technologies.^219,223^ For genetically engineered food crops to be fully commercialized, a country must have a comprehensive system that includes development, testing, commercialization, and often an extension education process for new crop policies and applications. However, in most SSA countries, the lack of technological capacity and technical expertise makes it challenging to implement these measures effectively ^219,223–225^ Additionally, products of genetic engineering are often seen as environmental threats, particularly if the resulting hybrids exhibit equal or greater vigor, fertility, and viability, posing a limitation to their adoption.^226^

Furthermore, activism groups known as anti-GM lobby groups, instead of fostering dialogue between proponents and opponents of genetically engineered technologies, often resort to public demonstrations. These protests, which may involve picketing along streets or at GM crop testing fields, aim to denounce modern biotechnology. Additionally, they use various communication media, such as radio and cell phones, to further their cause^224^ Sadikiel.^218^ This contributes to the reluctance and inability of many African countries, particularly those in SSA, to adopt genetic engineering technologies.

To ameliorate the barriers to the adoption of these technologies, potential strategies include public education and awareness campaigns to shape public opinion, incorporating scientific studies and findings into popular social media content, and organizing conferences for political leaders and decision-makers to collaboratively discuss biotechnology issues^220,224^; Sadikiel.^218^

## Prospect of Gene Editing in Sub-Saharan Africa

Sub-Saharan Africa presents prominent features and potential for gene editing applications in the improvement of staple crops with a specific emphasis on adoption within public breeding programs.^227^ Genome editing, as opposed to genetic modification (GM), is expected to become more widely accessible to smaller laboratories due to the supposed lower cost of its application making it a technology that “democratizes” molecular plant breeding.^205^ After missing out on the green revolution period to improve its agricultural system, Africa could benefit from these new technologies to finally boost its agriculture.^228,229^ In SSA, the adoption of genome editing for crop enhancement includes addressing challenges such as increasing food and nutrition security,^227,230^ reviving public breeding programs,^231^ eliminating persistent pests and diseases,^126,206,232,233^ and revolutionizing crop enhancement.^234,235,236^

## Conclusion

Sub-Saharan Africa is severely affected by climate change in terms of food security and agricultural output. The utilization of traditional plant breeding methodologies for the development of novel and enhanced varieties is arduous, laborious, and ineffective. The production of crop varieties with specific traits, including increased resistance to drought and high temperatures, is now possible due to advances in genome editing technology. The utilization of CRISPR/Cas9 technology in crops has recently increased gene editing applications for combating climate change. Genome editing has a significant position in enhancing agriculture in Africa. A multitude of researchers are now investigating the possibility of genome editing to enhance the development of crop types, to improve and promote an environmentally friendly agricultural system in Africa. Various crops, including disease-resistant banana, disease-resistant cassava, salinity tolerant rice, and drought tolerant wheat, are now being developed and are nearing completion for distribution in Africa. Nevertheless, the implementation of genome editing in Sub-Saharan Africa needs sufficient financial resources and supportive legislation to introduce genome editing solutions.

## Methodology Used in Writing the Review Paper

The review utilized a rigorous approach to assess Genome Editing in Sub-Saharan Africa: A Game-Changing Strategy for Climate Change Mitigation and Sustainable Agriculture. PubMed, Scopus, and Web of Science were searched for English-language studies published between 2010 and 2024. A standardized form extracted data on study characteristics, participants, interventions, outcomes, and quality. The Cochrane Risk of Bias tool was used to assess study quality. Findings were summarized through narrative synthesis due to the study of heterogeneity. Ethical considerations regarding off-target effects and long-term consequences were addressed. However, the review was limited to English-language studies.
